# Altered Mucus Glycosylation in Core 1 O-Glycan-Deficient Mice Affects Microbiota Composition and Intestinal Architecture

**DOI:** 10.1371/journal.pone.0085254

**Published:** 2014-01-09

**Authors:** Felix Sommer, Nina Adam, Malin E. V. Johansson, Lijun Xia, Gunnar C. Hansson, Fredrik Bäckhed

**Affiliations:** 1 The Wallenberg Laboratory and Sahlgrenska Center for Cardiovascular and Metabolic Research, Department of Molecular and Clinical Medicine, University of Gothenburg, Gothenburg, Sweden; 2 Mucin Biology Group, Department of Medical Biochemistry, University of Gothenburg, Gothenburg, Sweden; 3 Cardiovascular Biology Research Program, Oklahoma Medical Research Foundation, Oklahoma City, Oklahoma, United States of America; 4 Novo Nordisk Foundation Center for Basic Metabolic Research, Section for Metabolic Receptology and Enteroendocrinology, Faculty of Health Sciences, University of Copenhagen, Copenhagen, Denmark; Instutite of Agrochemistry and Food Technology, Spain

## Abstract

A functional mucus layer is a key requirement for gastrointestinal health as it serves as a barrier against bacterial invasion and subsequent inflammation. Recent findings suggest that mucus composition may pose an important selection pressure on the gut microbiota and that altered mucus thickness or properties such as glycosylation lead to intestinal inflammation dependent on bacteria. Here we used TM-IEC *C1galt* ^-/-^ mice, which carry an inducible deficiency of core 1-derived O-glycans in intestinal epithelial cells, to investigate the effects of mucus glycosylation on susceptibility to intestinal inflammation, gut microbial ecology and host physiology. We found that TM-IEC *C1galt* ^-/-^ mice did not develop spontaneous colitis, but they were more susceptible to dextran sodium sulphate-induced colitis. Furthermore, loss of core 1-derived O-glycans induced inverse shifts in the abundance of the phyla Bacteroidetes and Firmicutes. We also found that mucus glycosylation impacts intestinal architecture as TM-IEC C1galt^-/-^ mice had an elongated gastrointestinal tract with deeper ileal crypts, a small increase in the number of proliferative epithelial cells and thicker circular muscle layers in both the ileum and colon. Alterations in the length of the gastrointestinal tract were partly dependent on the microbiota. Thus, the mucus layer plays a role in the regulation of gut microbiota composition, balancing intestinal inflammation, and affects gut architecture.

## Introduction

The gastrointestinal tract harbours the most densely populated microbial ecosystem known encompassing more than 10^14^ bacteria, which outnumbers the cells of the human body by an order of magnitude. The gut microbiota resides in very close proximity to the host epithelium; however, despite this close association, the intestinal tract is normally healthy and free of inflammation primarily owing to a mechanical separation of host and microbial cells [1]–[3]. This separation is caused by the presence of several physical and biochemical barriers, the most important being gelatinous mucus, which covers the whole gastrointestinal tract [2]. Defects in the mucus layer are associated with intestinal inflammation [3], [4].

The mucus layer in the small intestine and colon is mainly made up of multimers of the mucin MUC2, a highly O-glycosylated protein of approximately 5200 amino acids and 2.5 MDa [5]. O-glycans contribute to about 80% of its mass and therefore mainly determine the physical mucus properties. O-glycosylation of MUC2 occurs post-translationally in the Golgi apparatus starting with the addition of the initial N-acetylgalactosamine (GalNAc) to the hydroxyl groups of serine and threonine in its two PTS domains (rich in proline, threonine and serine), where the larger is a stretch of about 2300 aa centrally located in MUC2 and rich in proline, threonine and serine [6]–[8]. The resulting GalNAcα-O-Ser/Thr structure is known as the Tn antigen and is normally not detectable as it is extended and branched by the action of other glycosyltransferases. The primary enzymes in this process are the core 1 β1,3-galactosyltransferase (C1galt1) in mice and core 3 β1,3-N-acetylglucosaminyltransferase (C3GnT) in humans [9], [10].

Mucus glycans not only protect the epithelial layer, but they also serve as an adhesion substrate for bacteria expressing adhesins and are a nutrient source for bacteria by hydrolysis through glycosidases [11], [12]. Thus, glycans could be an important factor in the selection of a beneficial microbiota and homeostasis. Indeed, glycosyltransferases have been shown to impact both intestinal inflammation and microbiota composition. In humans, loss of the α-1,2-fucosyltransferase FUT2, which is involved in the formation of ABO blood group antigens on the intestinal mucosa and in body fluids, also leads to an altered microbiota and increased susceptibility to infection and inflammatory disease such as Crohn's disease [13]. In mice, another blood-group-related glycosyltransferase β-1,4-N-acetylgalactosaminyltransferase 2 (B4galnt2) has been shown to affect gut microbiota composition [14] and thereby may affect susceptibility to intestinal inflammatory diseases.

Furthermore, mice fostered with milk of mothers deficient in the α2,3-sialyltransferase St3gal4 harbour an altered gut microbiota and are more resistant to dextran sodium sulphate (DSS)-induced colitis [15]. Finally, loss of core 3-derived O-glycans results in greater susceptibility to DSS-induced colitis [16] and loss of core 1-derived O-glycans has been reported to lead to spontaneous colitis [17]. Inflammation is in both models caused by altered mucus properties that abolish the separation of host epithelium and intestinal bacteria, thereby allowing bacterial penetration and overgrowth [4]. Disease development can be ameliorated by antibiotic treatment [17].

Together, these data indicate a causal role for the gut microbiota in the induction of colitis in susceptible hosts. However, it remains to be determined if the altered mucus in these models selects for a more colitogenic microbiota that then causes inflammation. Here we used mice that carry an inducible deficiency of core 1-derived O-glycans in intestinal epithelial cells to investigate if mucus glycosylation affects gut microbial ecology and thereby host physiology as well as susceptibility to intestinal inflammation.

## Materials and Methods

### Mice

*C1galt1* ^f/f^;Villin-Cre-ER^T2^ (TM-IEC *C1galt1* ^-/-^) mice have been previously described [17] and were rederived as germ-free at Taconic. Germ-free mice were maintained in flexible film isolators. A conventional cohort was established by colonizing germ-free TM-IEC *C1galt1* ^-/-^ mice with caecal flora of C57BL/6 mice and bred for at least three generations in our animal facility under specific pathogen-free conditions. All mice were housed under a 12-h light cycle and fed autoclaved chow diet ad libitum (Labdiet, St Louis, Missouri, USA). TM-IEC *C1galt1* ^-/-^ mice were bred using heterozygous setup for Villin-Cre-ER^T2^ alleles to facilitate littermate controls. Excision of the *C1galt1* gene was induced by i.p. injection of 1 mg tamoxifen for five consecutive days. Experiments were initiated after a further ten days. All experiments were performed using protocols approved by the Gothenburg Animal Ethics Committee (339-2012, 280-2012 and 281-2012).

### Histology

For histological analyses, we used 8-9-week-old female mice that were killed by cervical dislocation. Intestinal specimens were harvested and fixed in methacarn (60% dry methanol, 30% dry chloroform, 10% glacial acetic acid) for 1-2 weeks at room temperature prior to paraffin embedding and sectioning. Sections were stained with haematoxylin/eosin (HE) for morphology, Alcian blue/periodic acid-Schiff (AB-PAS) stain for glycan composition, and Mab anti-Tn antibody (clone 5F4, [18]) with rat-anti-mouse-FITC antibody (BD Pharmingen) for detection of the Tn antigen. Muscle was visualized using rabbit-anti-smooth muscle actin antibody coupled to Cy3 fluorochrome (Sigma) and proliferative cells were stained using rabbit-anti-Ki-67 (Thermo Scientific) antibody and Vectastain Elite ABC kit (Rabbit IgG, Vector labs). HE and HOECHST were used as counterstaining for enzymatic and fluorescent detection, respectively. Villus length and crypt depth were assessed using HE-stained sections. Microscopy measurements were performed using Zeiss Axiovision LE v4.8 on sections of at least five mice per genotype with each 15 individual scores. Number of lamina propria cells was counted for 5–7 mice. For ileum five complete villi and for colon ten areas between two complete crypts per mouse were counted.

### DSS treatment

Colonic inflammation was induced in 9–14-week-old male and female mice by administration of 3% DSS (TdB TdB Consultancy, Uppsala, Sweden) for five days in drinking water followed by a five-day recovery period before killing the mice by cervical dislocation [19]. Weight and faeces of the mice were monitored daily. Colitis severity was assessed by calculating the disease activity index (DAI) combining the scores of weight loss, stool consistency and faecal bleeding [20]. Briefly, the scoring system was as follows: weight loss: 0 =  no loss, 1 = 1–5%, 2 = 5–10%, 3 = 10–20%, 4 = >20%; stool: 0 = normal, 2 =  loose stool, 4 =  diarrhoea; and bleeding: 0 =  no blood, 2 =  cryptic blood (Hemoccult positive, Hemoccult II; Beckman Coulter), and 4 =  heavy bleeding. Interleukin (IL)-1β and tumor necrosis factor (TNF)α levels were measured in protein lysates from colon tissues of TM-IEC *C1galt1* ^-/-^ and wild type mice using Mouse Basic Kit FlowCytomix with IL-1β and TNFα simplex (eBioscience) according to the manufacturer's instructions.

### Microbiota analysis by 16S rDNA pyro-sequencing

Total genomic DNA was extracted from 100–200 mg snap frozen caecum of 8–9-week-old female mice as described previously [21]–[23]. The V2–V3 region of the 16S rDNA gene was amplified using barcoded primers. Purified amplicons were pooled and concentration was adjusted to 20 ng/µl for 454 pyrosequencing. Sequence data were analyzed using MacQIIME package v1.6 (http://www.wernerlab.org/software/macqiime, [24]). Briefly, sequencing reads were trimmed and mapped onto the different samples using the barcode information. Next, reads were assigned to operational taxonomic units (OTUs) using 97% identity, representative OTUs were picked and taxonomy assigned. Quality filtering was performed by removing chimeric sequences using ChimeraSlayer and by removing singletons. Relative abundance was calculated by dividing the number of reads for an OTU by the total number of sequences in the sample. Unifrac alpha and beta diversity were calculated and phylogeny constructed using UPGMA (Unweighted Pair Group Method with Arithmetic Mean). Significance of differences in abundances of various taxonomic units was calculated using t-test and false discovery-rate correction in R program.

## Results

### Loss of core 1-derived O-glycans in adult mice increases susceptibility to DSS-induced intestinal inflammation

**Figure 1:**
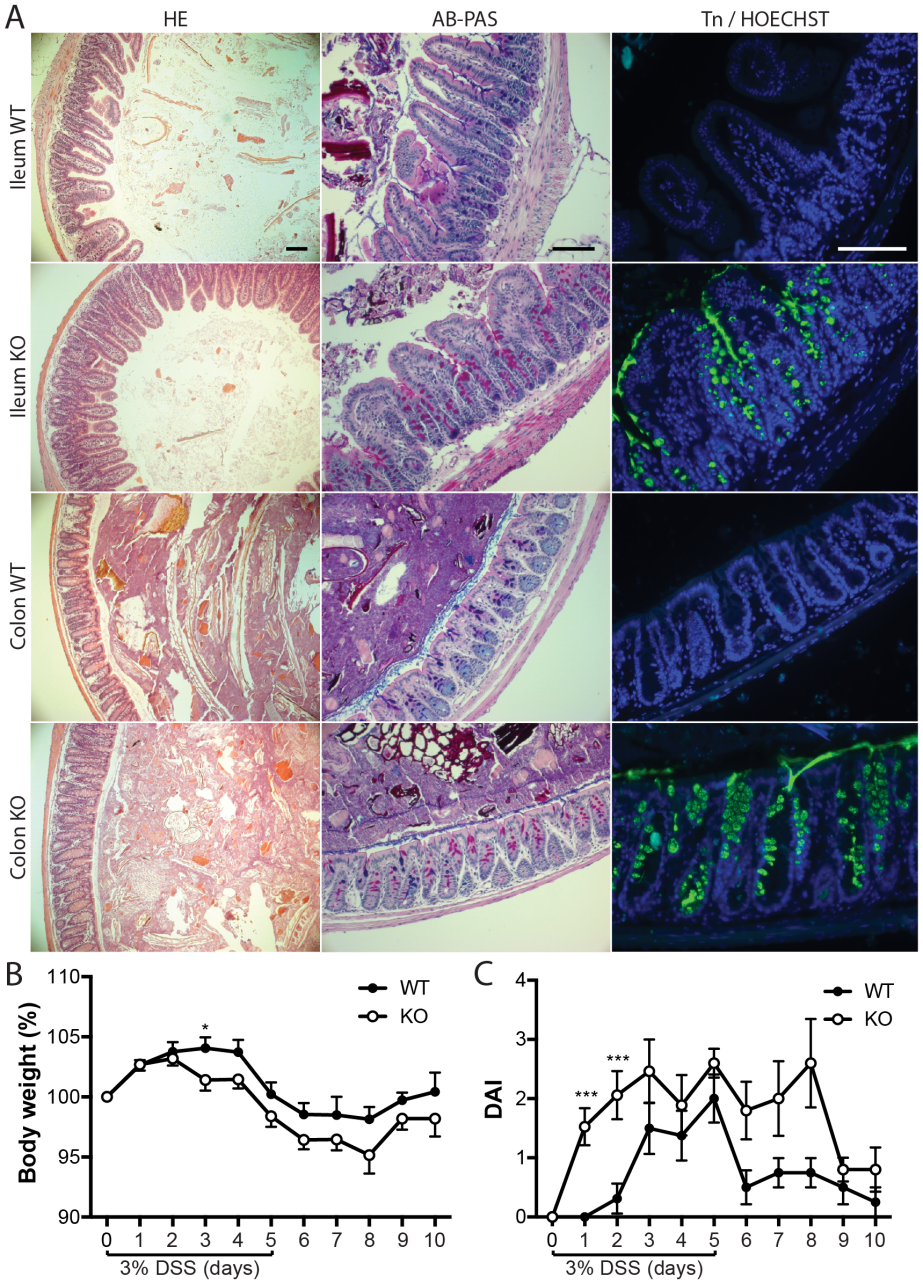
TM-IEC *C1galt* ^-/-^ mice are not spontaneously colitic but more susceptible to DSS-induced intestinal inflammation. (A) Histological analysis by HE, AB-PAS and Tn antibody staining of sections taken ten days post knockout induction from ileal and colonic tissue of wild type (WT) and TM-IEC *C1galt* ^-/-^ (KO) mice. Scale bars indicate 100 µm. (B) Body weight and (C) DAI during DSS treatment and recovery period (n = 16 per genotype for day 0, two mice sacrificed every day during DSS treatment; Data shows mean ± SEM; ** p<0.01, *** p<0.001).

In several genetic mouse models used to study intestinal inflammation conditional transgenic mice are used. However, the mice are born with the respective defect causing inflammation, which may affect microbial composition per se, and thus this strategy is suboptimal to elucidate selection effects of host structures on the gut microbiota. Here we used recently developed TM-IEC *C1galt* ^-/-^ mice, which carry an inducible deficiency of core 1-derived O-glycans in intestinal epithelial cells in which the floxed *C1galt1* gene is excised only after induction of the otherwise inert Cre recombinase by administration of tamoxifen. In contrast to a previous study in which TM-IEC *C1galt* ^-/-^ mice developed intestinal inflammation ten days after induced loss of core 1-derived O-glycans [17], TM-IEC *C1galt* ^-/-^ mice in our animal facility did not develop spontaneous colitis or show any signs of inflammation ten days (Fig. 1A, left panels) or even up to 26 weeks after knockout induction. This was not caused by an inability to induce the knockout allele since the TM-IEC *C1galt* ^-/-^ mice showed an increased ratio of cells producing neutral versus acidic carbohydrates (Fig. 1A, middle panels) and a clear removal of core 1-derived O-glycans exposing the Tn antigen after administration of tamoxifen (Fig. 1A, right panels). Furthermore, TM-IEC *C1galt* ^-/-^ mice displayed an altered mucus glycosylation pattern compared with the Cre-negative wild type littermates (data not shown, in agreement with a recent report on the constitutively deleted C1galt1 [10]).

Importantly, the TM-IEC *C1galt* ^-/-^ mice responded more quickly and with greater severity to treatment with DSS. After three days of DSS treatment, TM-IEC *C1galt* ^-/-^ mice had a significantly lower body weight than the wild type littermate controls and were unable to recover from this weight difference throughout the experiment (Fig. 1B). Furthermore, disease activity index (DAI; the combined score of weight loss, stool consistence and faecal bleeding) was higher in TM-IEC *C1galt* ^-/-^ mice on the first two days of the DSS treatment (Fig. 1C). Blood in the faeces was the main contributor to the DAI with only minor changes in faecal consistency. TM-IEC *C1galt* ^-/-^ mice seemed to recover more slowly from the DSS-induced colitis. Faecal blood could still be detected three days after the DSS treatment was stopped in TM-IEC *C1galt* ^-/-^ mice whereas the wild type controls recovered within one day (Fig. 1C). In addition, the colons of TM-IEC *C1galt* ^-/-^ mice had a slight but non-significant increase in IL-1β and a significantly elevated level of TNFα compared with those of wild type mice (Fig. S1). Taken together, these data indicate that although the TM-IEC *C1galt* ^-/-^ mice did not develop spontaneous colitis in our study, they were more susceptible to DSS-induced gut inflammation.

### Mucus glycosylation has a minor influence on gut microbiota composition

**Figure 2:**
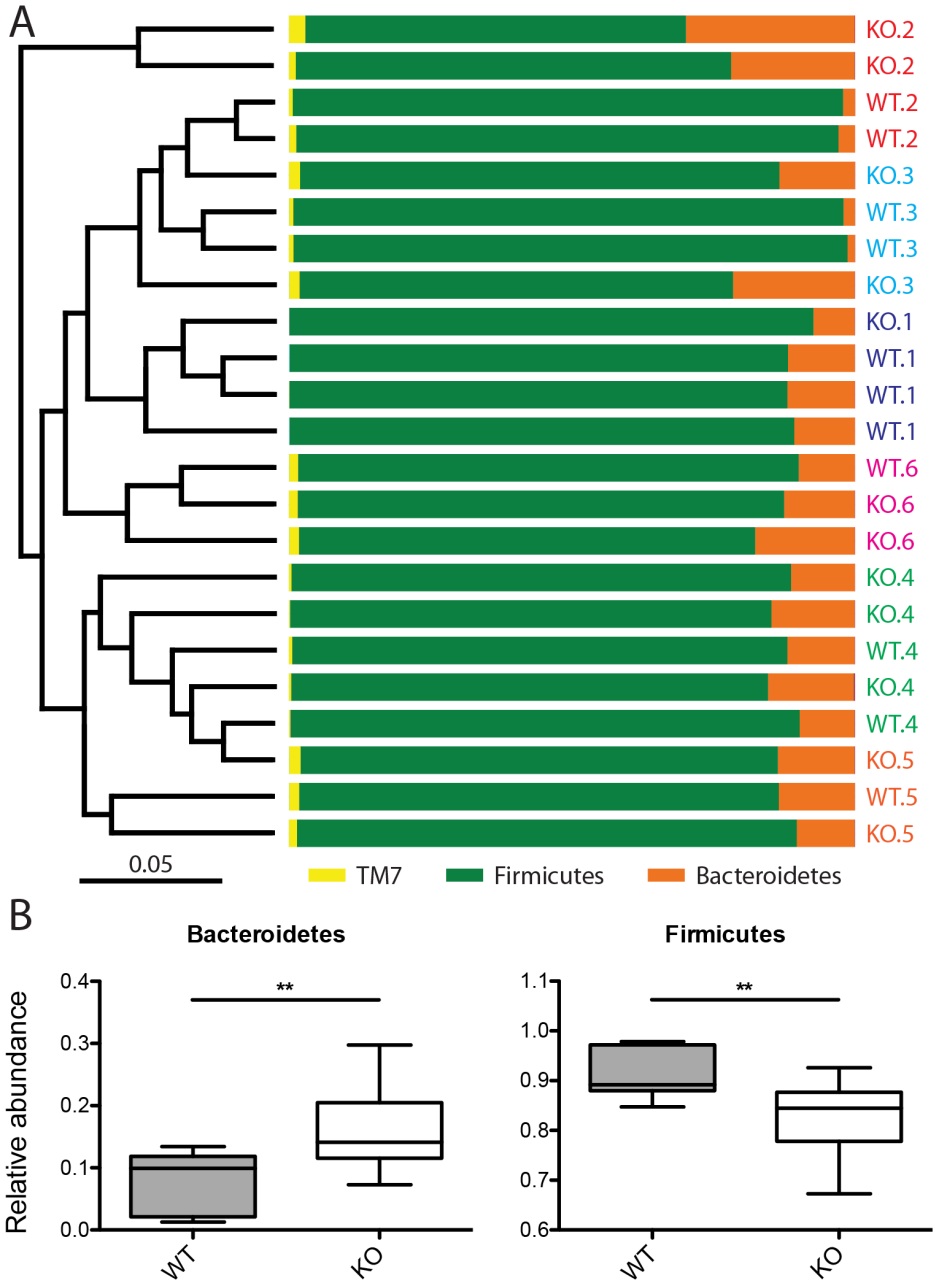
Mucus glycosylation influences gut microbiota composition. (A) Microbiota composition and phylogenetic tree on phylum level. Labels indicate genotype and cage number. Samples are colour coded according to cage number. (B) Relative abundance of Bacteroidetes and Firmicutes in wild type (WT) and TM-IEC *C1galt* ^-/-^ (KO) mice (Data shows mean ± SEM; ** p≤0.01, *** p≤0.001).

To test the hypothesis that mucus glycosylation plays a role in selecting the microbiota and maintaining homeostasis, we analyzed the microbial composition of TM-IEC *C1galt* ^-/-^ mice and their wild type littermates. When comparing the overall microbial composition, we found that the mice grouped primarily according to cage and within a cage the mice grouped secondarily according to genotype, indicating that loss of core 1-derived O-glycans induced subtle alterations in the microbiota (Fig. 2A). Abundance of the phylum Bacteroidetes was increased whereas that of Firmicutes was reduced in TM-IEC *C1galt* ^-/-^ mice (Fig. 2B). These phylum differences were reflected by lower taxonomic levels as well with, for example, classes bacteroidia and clostridia showing the same trends (Fig. S2, S3). However, on a species level, only one operational taxonomic unit (OTU) differed significantly among TM-IEC *C1galt* ^-/-^ and wild type mice (Fig. S2F), indicating rather broad but subtle differences in microbial abundance caused by loss of core 1-derived O-glycans. Moreover, no differences in alpha diversity (the number of microbial species in a sample) were detected (Fig. S4). In summary, in the absence of any detectable inflammation, loss of core 1-derived O-glycans seems to exert a subtle selection effect on the gut microbiota.

### Lack of core 1 mucus glycosylation alters the intestinal architecture

**Figure 3:**
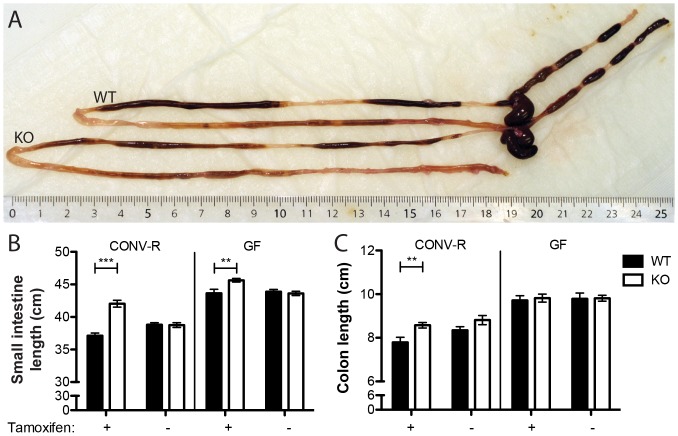
Lack of core 1-derived mucus O-glycosylation alters gut length. (A) Representative gastrointestinal tracts of wild type (WT) and TM-IEC *C1galt* ^-/-^ (KO) mice. (B) Length of the small intestine and colon of wild type (WT) and TM-IEC *C1galt* ^-/-^ (KO) mice raised in the presence (CONV-R) and absence of bacteria (GF) as well as with or without injection of tamoxifen to induce Cre recombinase-mediated loss of core 1-derived mucus O-glycosylation (n = 7-16 mice per group; Data shows mean ± SEM; ** p<0.01, *** p<0.001).

The most obvious phenotypic feature of TM-IEC *C1galt* ^-/-^ mice was an extension in the length of gastrointestinal tract. Both the small and large intestines of the TM-IEC *C1galt* ^-/-^ mice were about 10% longer than those of wild type controls (Fig. 3A–C). Notably, the effect on gut length was not seen in the absence of tamoxifen activation of the Cre recombinase and thus dependent on the core 1-derived O-glycans (Fig. 3B, C). Regardless of the genotype, the length of the gastrointestinal tract in germ-free mice was longer than that of conventional counterparts. Furthermore, in the small intestine the effect on gut length in the TM-IEC *C1galt* ^-/-^ mice seemed to be partly dependent on the microbiota as the difference in length between knockout and wild type mice decreased from 13.2% (p<0.0001) to 4.6% (p = 0.0039) in conventional and germ-free setting, respectively. In contrast, in the colon the effect on gut length seemed to be solely dependent on the microbes (decreased from 10.1%, p = 0.006 to 1.1%, p = 0.72 in the conventional and germ-free setting, respectively; Fig. 3C).

**Figure 4:**
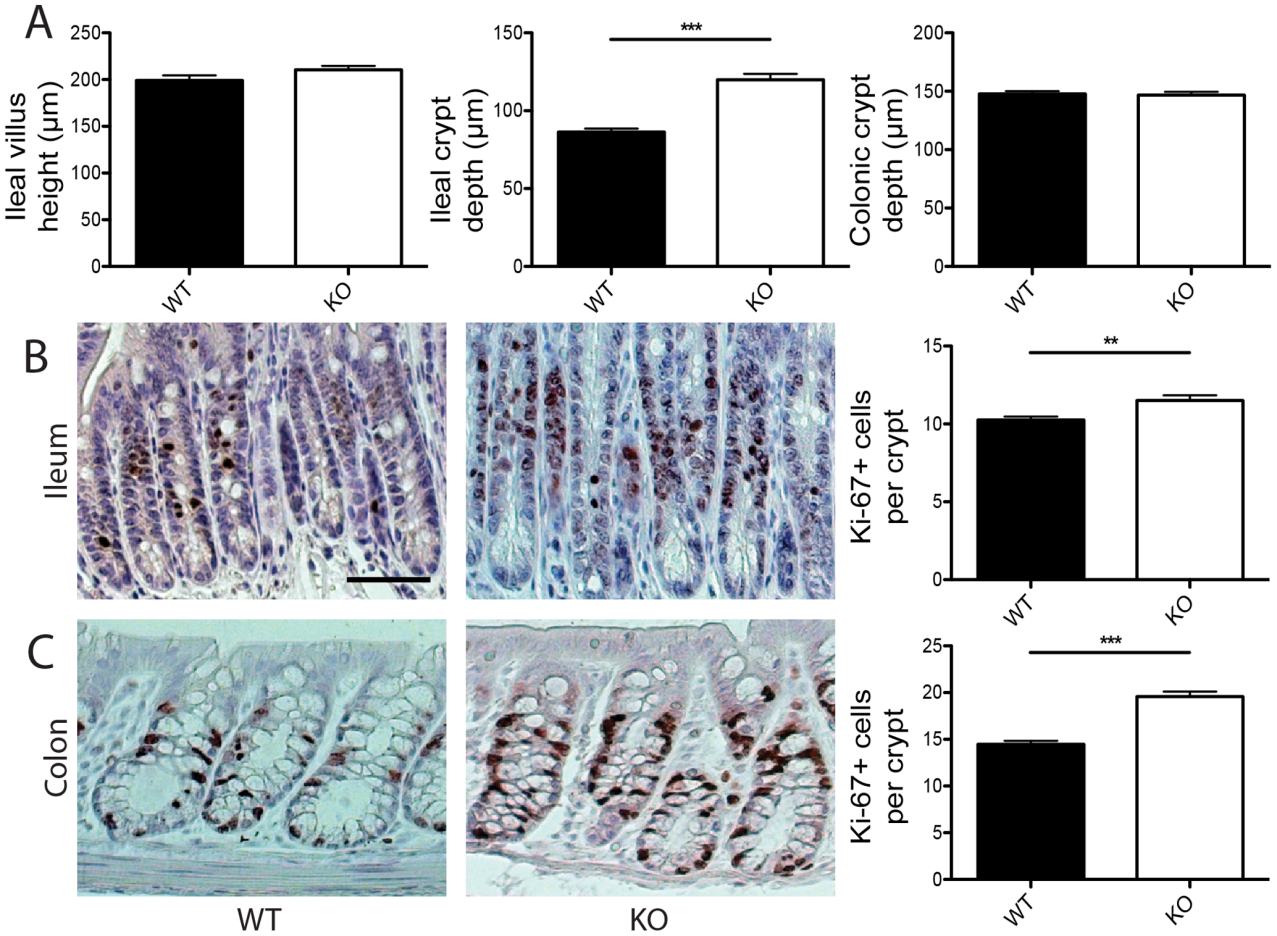
Intestinal architecture and proliferation are altered in TM-IEC *C1galt* ^-/-^ mice. (A) Ileal villus height and ileal and colonic crypt depth of wild type (WT) and TM-IEC *C1galt* ^-/-^ (KO) mice. (B–C) Ki-67 stainings and quantification of sections from ileal (B) and colonic (C) tissue of wild type (WT) and TM-IEC *C1galt* ^-/-^ (KO) mice. For quantifications n = 5–7 mice were scored. Scale bar indicates 50 µm (B–C). Data shows mean ± SEM; ** p<0.01, *** p<0.001.

**Figure 5:**
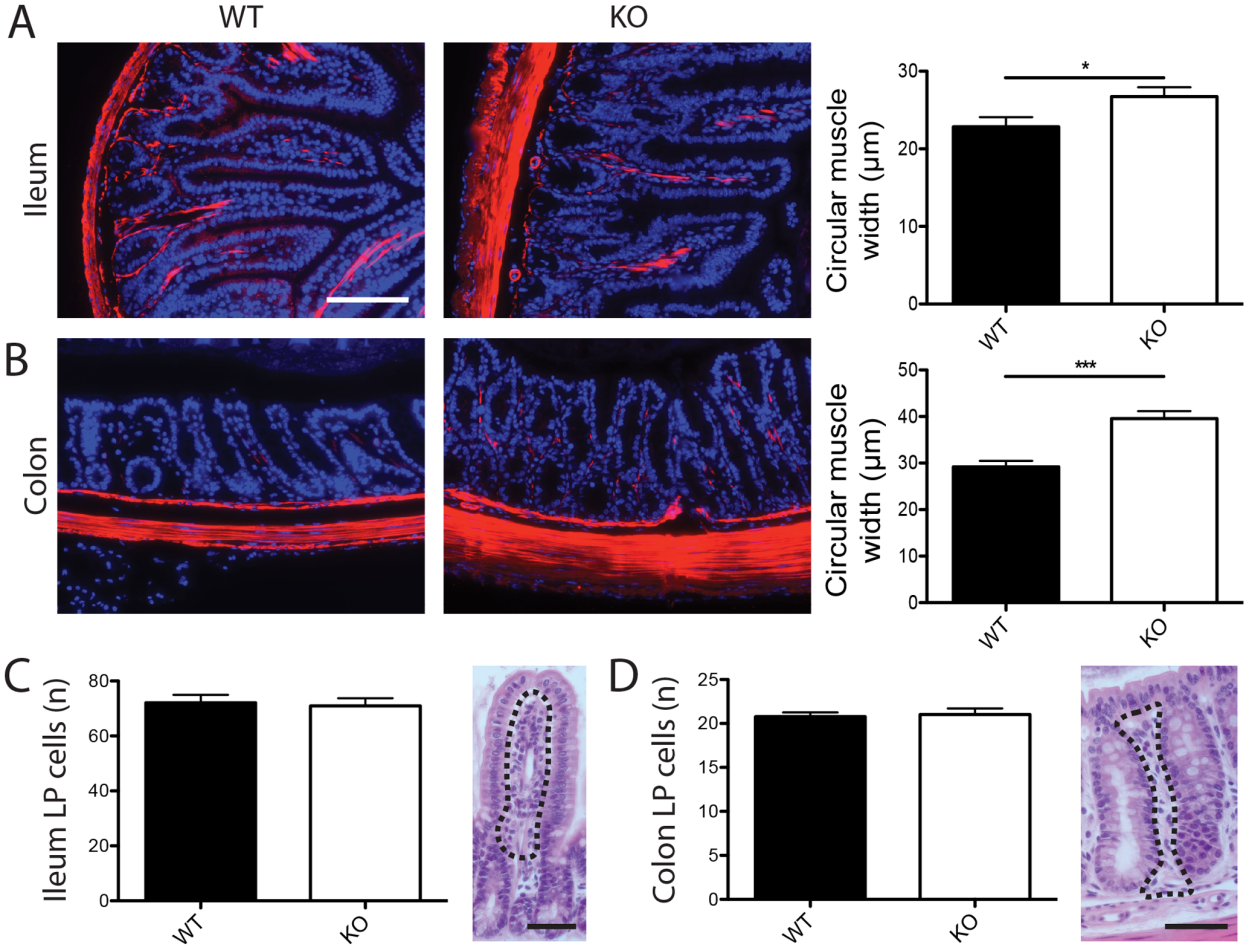
Intestinal muscle width is increased in TM-IEC *C1galt* ^-/-^ mice in absence of immune cell infiltration. (A–B) Smooth muscle actin stainings and quantification of sections from ileal (A) and colonic (B) tissue of wild type (WT) and TM-IEC *C1galt* ^-/-^ (KO) mice. (C–D) Quantification and representative image of ileal (C) and colonic (D) lamina propria cells. LP - lamina propria. The dotted lines represent the areas used for scoring. For quantifications n = 5–7 mice were scored. Scale bars indicate 50 µm. Data shows mean ± SEM; * p<0.05, *** p<0.001.

In addition to gut length, structural architecture of the gastrointestinal tract was also altered by loss of core 1-derived O-glycans. Although length of the ileal villi and depth of the colonic crypts did not differ between TM-IEC *C1galt* ^-/-^ and wild type mice, ileal crypts were deeper in TM-IEC *C1galt* ^-/-^ mice (Fig. 4A). We also observed small but significant increases in the number of Ki-67 positive cells both in ileal and colonic crypts of TM-IEC *C1galt* ^-/-^ mice (Fig. 4B, C), indicating slightly increased proliferation of intestinal epithelial cells. Furthermore, the circular muscle layers both in the ileum and colon were thicker in TM-IEC *C1galt* ^-/-^ mice (Fig. 5A, B). However, numbers of immune cells in the lamina propria were not altered in either the ileum or colon of TM-IEC *C1galt* ^-/-^ mice compared with wild type controls (Fig. 5C, D).

## Discussion

We aimed to investigate if mucus glycosylation has a selective effect on gut microbial ecology and thereby might impact susceptibility to intestinal inflammation and host physiology. Therefore, we used TM-IEC *C1galt* ^-/-^ mice which carry an inducible deficiency of core 1-derived O-glycans in intestinal epithelial cells as a model since it allows us to discriminate between selection effects of host structures in the adult mouse from those present when mice are born and raised with the respective defects. We found that TM-IEC *C1galt* ^-/-^ mice did not show any detectable signs of inflammation, but were more susceptible to DSS-induced colitis. This is in contrast to a previous study of TM-IEC *C1galt* ^-/-^ mice kept in another animal house where they spontaneously developed intestinal inflammation which was dependent on the microbiota ten days after induced loss of core 1-derived O-glycans [17]. Thus, the discrepancy between our and previous findings is probably due to different housing conditions and microbiota in the animal facilities. Presumably, the gut microbiota composition of the TM-IEC *C1galt* ^-/-^ mice rederived and bred in our facility has a flora that is less colitogenic. This is also observed for IL-10^-/-^ mice that normally have spontaneous colitis, but in our animal house only show minor inflammation [4], [25]. An impact of housing on experimental phenotypes has been observed previously in other models. For example, C56BL/6 mice bred by Taconic Farms harbour more lamina propria lymphocytes than those bred by Jackson Laboratory and only caecal flora from Taconic mice is able to induce lamina propria lymphocyte formation in colonized germ-free mice [26]. Similarly, differential susceptibility to streptozotocin-induced diabetes in mice from Taconic Farms, Jackson Laboratory or Charles River Laboratories has been observed [27], [28]. Together, these findings highlight the importance of the microbiota in the development of several inflammatory or metabolic diseases and the need to keep experimental setups including housing conditions and microbiota as stable as possible to facilitate comparisons among studies performed in different animal facilities.

A number of recent publications highlighted the interaction between the intestinal mucus layer, its glycosylation and the microbiota for intestinal homeostasis [29]–[32]. Here, we made two observations that suggest a microbial role in intestinal homeostasis. (i) Loss of core 1-derived O-glycans, in the absence of inflammation, induced subtle gut microbial alterations, for example inverse shifts in the abundance of the phyla Bacteroidetes and Firmicutes, indicating that glycosylation of the intestinal mucus layer has a selective capacity on microbial ecology. Similarly, other immune components have previously been shown to modulate the microbiota [33]. (ii) The gastrointestinal tract was elongated in TM-IEC *C1galt* ^-/-^ mice and these alterations were partly microbially dependent. The small changes in intestinal architecture may thus potentially result from the altered microbial ecology.

We also observed other intestinal changes in TM-IEC *C1galt* ^-/-^ mice compared with wild type controls, namely increased ileal crypt depth, a small increase in the number of proliferating epithelial cells, and a thicker circular muscle layer in the ileum and colon of TM-IEC *C1galt* ^-/-^ mice. This last observation might indicate differential muscle contraction in the TM-IEC *C1galt* ^-/-^ mice and could potentially contribute to the observed differences in length of the gastrointestinal tract of TM-IEC *C1galt* ^-/-^ mice.

Notably, it has previously been shown that the gut microbiota affects several aspects of host physiology within and outside of the gastrointestinal tract including organ morphogenesis and tissue homeostasis [1]. In Drosophila, the gut microbiota modulates length of the gastrointestinal tract along with proliferation and differentiation of intestinal epithelial cells [34]. In mice, proliferation of epithelial cells is reduced in the small intestine of germ-free compared to conventionally raised mice [35]–[37]. Furthermore, the gut microbiota influences intestinal architecture including villus and crypt morphology as well as remodelling of the intestinal vascular system [38], [39]. Altogether these observations suggest that the gut microbiota represents an important environmental factor in the regulation of intestinal homeostasis and physiology.

An altered intestinal architecture without major infiltration of immune cells is reminiscent of some of the features of the human disease irritable bowel syndrome (IBS) [40]. Furthermore, both hypertrophy of the jejunal muscle layer [41] and alterations in faecal microbiota composition were reported to be associated with IBS [42]. Further studies are required to determine if TM-IEC *C1galt* ^-/-^ mice could represent an animal model for IBS.

Taken together, mucus glycosylation seems to be important not only for protection and lubrication of the gastrointestinal tract, but also has selective effects on the composition of the resident gut microbiota and small effects on the structure of the intestine. The small alterations in mucus properties, microbiota composition and slightly increased epithelial proliferation in TM-IEC *C1galt* ^-/-^ mice could indicate a skewed intestinal homeostasis with altered regenerative response and intestinal architecture in the absence of inflammation. This could potentially suggest a number of physiological defence actions preceding an overall inflammatory response with infiltrating immune cells.

## Supporting Information

Figure S1 **Levels of pro-inflammatory cytokines IL-1β and TNFα in colon of TM-IEC** ***C1galt^-/-^*** **and wild type mice.** Proteins were isolated from colonic tissue of wild type (WT) and TM-IEC *C1galt* ^-/-^ (KO) mice and IL-1β and TNFα measured with n = 4 mice per group. Data shows mean ± SEM; * p<0.05.
(TIF)

Figure S2 **Taxa differentially abundant among wild type and TM-IEC** ***C1galt*** **^-/-^ mice.** Data shows mean ± SEM; * p<0.05, ** p<0.01.
(TIF)

Figure S3 **Abundance overview of microbial phyla on class, order, family and genus level.** Labels indicate genotype and cage number. Samples are colour coded according to cage number.
(TIF)

Figure S4 **Microbial alpha-diversity in wild type and TM-IEC** ***C1galt*** **^-/-^ mice.**
(TIF)
