# Supplementary figures and images for: Altered Mucus Glycosylation in Core 1 O-Glycan-Deficient Mice Affects Microbiota Composition and Intestinal Architecture
Source: PLoS One. 2014 Jan 9;9(1):e85254. doi: 10.1371/journal.pone.0085254 (PMC3887012; doi:10.1371/journal.pone.0085254)

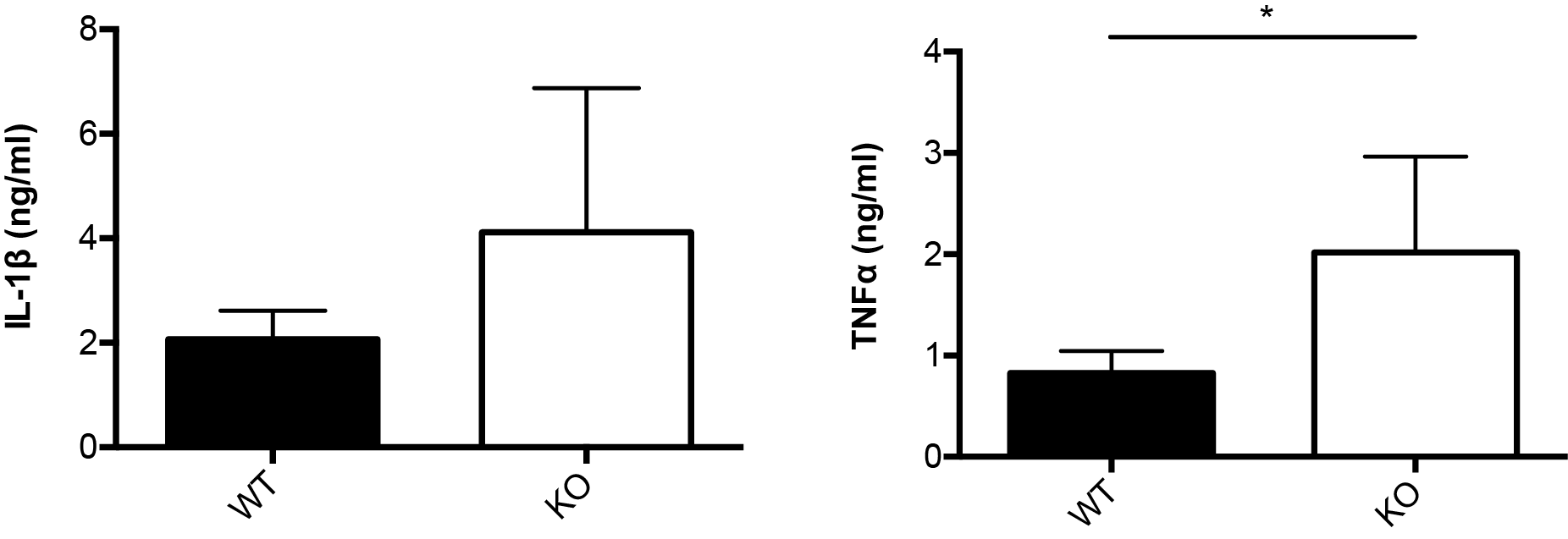

Supplement: Figure S1 — Levels of pro-inflammatory cytokines IL-1β and TNFα in colon of TM-IEC C1galt-/- and wild type mice. Proteins were isolated from colonic tissue of wild type (WT) and TM-IEC C1galt -/- (KO) mice and IL-1β and TNFα measured with n = 4 mice per group. Data shows mean ± SEM; * p<0.05. (TIF) [file pone.0085254.s001.tif]

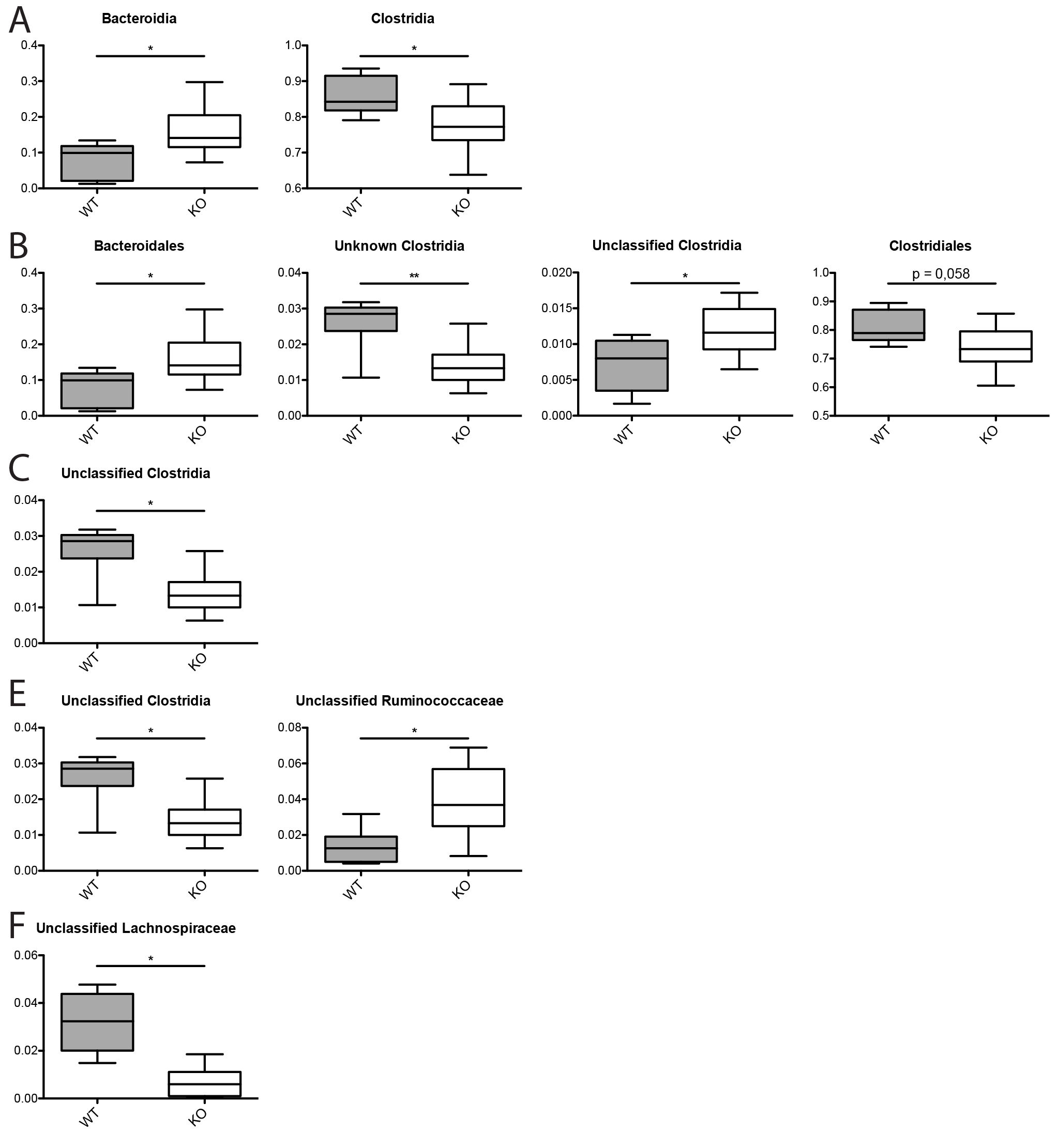

Supplement: Figure S2 — Taxa differentially abundant among wild type and TM-IEC C1galt -/- mice. Data shows mean ± SEM; * p<0.05, ** p<0.01. (TIF) [file pone.0085254.s002.tif]

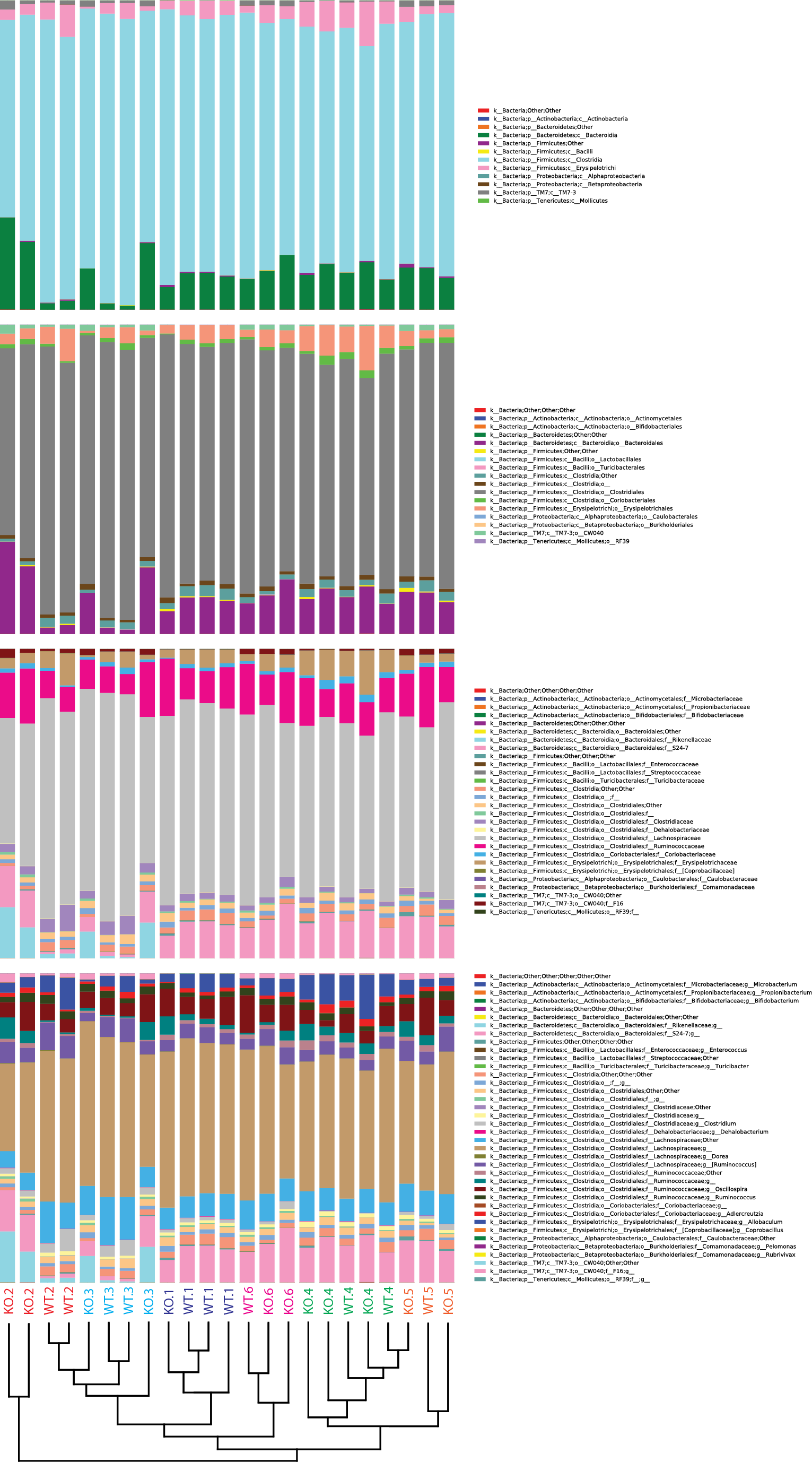

Supplement: Figure S3 — Abundance overview of microbial phyla on class, order, family and genus level. Labels indicate genotype and cage number. Samples are colour coded according to cage number. (TIF) [file pone.0085254.s003.tif]

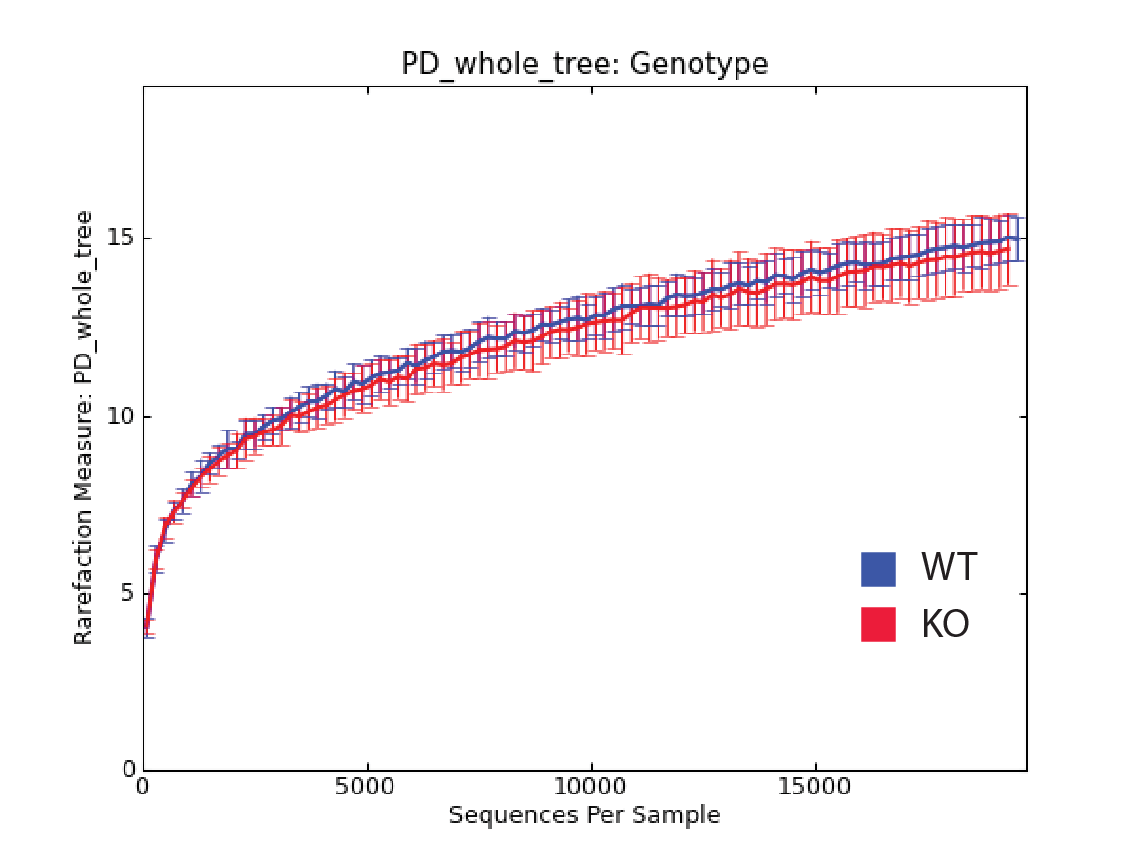

Supplement: Figure S4 — Microbial alpha-diversity in wild type and TM-IEC C1galt -/- mice. (TIF) [file pone.0085254.s004.tif]
